# Sense of coherence and its relationship to participation, cancer-related fatigue, symptom burden, and quality of life in women with breast cancer participating in the OptiTrain exercise trial

**DOI:** 10.1007/s00520-020-05378-0

**Published:** 2020-03-05

**Authors:** Anouk E Hiensch, Kate A Bolam, Sara Mijwel, Anne M May, Yvonne Wengström

**Affiliations:** 1 Julius Center for Health Sciences and Primary Care, University Medical Center Utrecht, Utrecht University, Utrecht, The Netherlands; 2 Department of Neurobiology, Care Sciences and Society, Karolinska Institutet, Stockholm, Sweden; 3 Theme Cancer, Karolinska University Hospital, Stockholm, Sweden

**Keywords:** Sense of coherence, Physical exercise, Adherence, Breast cancer, Cancer-related fatigue, Quality of life, Chemotherapy

## Abstract

**Purpose:**

This study examined the Sense of Coherence (SOC) of patients participating in the randomized controlled ‘Optimal Training for Women with Breast Cancer’ (OptiTrain) study and assessed how patient characteristics were associated with SOC. Secondary aims were to assess the association between SOC and patients’ participation in this study and to determine whether SOC moderates the effect of the 16-week exercise intervention on fatigue, quality of life (QoL), and symptom burden in women with breast cancer undergoing chemotherapy.

**Methods:**

Modified Poisson regression analyses were conducted to determine the relative risk of weak-normal SOC versus strong SOC in terms of exercise session attendance, study and intervention dropout, and long absence rates. Analyses of covariance were performed to assess whether SOC moderated the effect of the exercise intervention (*p*_interaction_ ≤ 0.10).

**Results:**

Two hundred and forty women with early breast cancer (mean age 53 ± 10) participated in the OptiTrain study. Women with strong SOC reported less fatigue, lower symptom burden, and higher QoL. Women with weak-normal SOC were significantly more likely to drop out from the OptiTrain study and tended to have slightly poorer exercise session attendance. Women with breast cancer and weaker SOC benefitted as much from the exercise intervention, in terms of fatigue and QoL, as those with stronger SOC (*p*_interaction_ > 0.10).

**Conclusions:**

Strong SOC appears to be associated with a more positive subjective state of health. Women with weak-normal SOC may need additional support to encourage participation and adherence in exercise trials. Assessing SOC may assist clinicians to identify and provide extra support for participants with weak SOC, who may be less inclined to participate in exercise programs.

**Electronic supplementary material:**

The online version of this article (10.1007/s00520-020-05378-0) contains supplementary material, which is available to authorized users.

## Background

An increasing number of studies have examined the effects of exercise on cancer and treatment-related side effects. Conclusions from meta-analyses of randomized controlled trials (RCTs) support the notion that exercise interventions, delivered during adjuvant systemic cancer treatment, can significantly reduce cancer-related fatigue [1, 2], and improve physical fitness [1, 3, 4] and quality of life (QoL) [3, 5].

The efficacy of an exercise intervention largely depends on patients’ participation, attrition, and exercise session attendance rates. In general, a limited proportion of eligible patients take part in exercise trials. In addition, a major problem in RCTs is loss to follow-up [6]. It can be argued that patients who choose to volunteer for exercise trials are not completely representative of the target population (i.e. all patients with cancer undergoing chemotherapy). As a consequence of selective participation and dropout, the observed intervention effect might underestimate or overestimate the true effect in the target population.

Modifiable (e.g. psychosocial) as well as non-modifiable (e.g. demographical) participant characteristics might explain participation, attrition, and attendance rates in exercise interventions. Insight into relevant participant characteristics enables us to optimize attendance in randomized controlled exercise trials. It also allows us to have a better understanding of the bias that might be introduced by selective drop-out. Additionally, it provides information about how we might adapt future exercise intervention studies to identify and make contact with those patients who are less inclined to participate, and how to motivate those patients who are more likely to drop out from the intervention; eventually, this may facilitate successful implementation of exercise interventions.

In order to improve our understanding of participation, drop-out, and attendance in exercise interventions, behavioural models of determinants that might explain exercise behaviour should be taken into account [7, 8]. Specifically, one theoretical construct, a sense of coherence (SOC) can be potentially helpful in explaining exercise behaviour in patients with cancer. According to Antonovsky’s salutogenesis theory, the SOC scale appears to be relatively steady over time and refers to an enduring, though dynamic, attitude that one’s internal and external environment are structured, consistent, and understandable [9, 10]. The SOC questionnaire measures how people cope with stressful situations to both maintain and improve their health [9]. Strong SOC implies successful management of stressors to maintain and improve an individuals’ wellbeing. Correspondingly, those with stronger SOC may more actively pursue healthy activities [11] and may therefore more actively engage in exercise interventions. In summary, it can be assumed that SOC may affect participation, drop-out, and attendance in exercise interventions.

Accumulating evidence supports a direct and indirect association between strong SOC and perceived health (i.e. QoL, symptom burden and distress) [11]. However, it is unclear whether patients with strong SOC also benefit more from health-promoting interventions such as exercise interventions.

In the randomized controlled ‘Optimal Training for Women with Breast Cancer’ (OptiTrain) study, we demonstrated that combined resistance and aerobic high-intensity interval training during chemotherapy had a significant beneficial effect on cancer-related fatigue [12]. The main objective of the present study was to examine the SOC of patients participating in the OptiTrain trial and to assess how patient characteristics are associated with SOC. Secondary aims were (1) to assess the association between SOC and patients’ exercise session attendance, study and intervention dropout, and long-term absence during the intervention period, and (2) to determine whether SOC moderates the effect of the 16-week exercise intervention on fatigue, QoL, and symptom burden in women with breast cancer undergoing chemotherapy.

## Methods

### Study design and participants

A detailed description of the OptiTrain study design has been published previously [13]. In short, this 16-week in-clinic randomized controlled exercise trial was conducted at the Karolinska University Hospital, Södersjukhuset, Stockholm, Sweden, between March 2013 and August 2016. Inclusion criteria were women (aged 18–70 years) diagnosed with I–IIIa stage breast cancer, scheduled for adjuvant chemotherapy. Exclusion criteria were as follows: advanced disease, heart or lung disease, cognitive dysfunction, other health problems that would prevent safe participation in the exercise testing or training as determined by their medical doctor, or not being able to understand the Swedish language. The referring oncologist invited all consecutive patients, who met the inclusion criteria, to participate in the study. Interested patients filled out a questionnaire about their cardiovascular health history and underwent a resting echocardiogram. If no relevant health issues were identified, the patients were deemed fully eligible for participation in the study and written informed consent was obtained. Participants were randomly allocated to either moderate-intensity aerobic and high-intensity interval training (AT-HIIT), resistance and high-intensity interval training (RT-HIIT), or usual care (UC) following a 1:1:1 ratio using a computer-generated program.

Ethical permission was obtained from the Regional Ethical Review Board in Stockholm (Dnr 2012/1347-31/1, 2012/1347-31/2, 2013/7632-32 and 2014/408-32).

### Intervention

Participants in the exercise intervention groups started the exercise training program 3 days after the second chemotherapy session. Participants were asked to attend 60-min exercise sessions, twice-weekly, on non-consecutive weekdays for 16 weeks. Exercise sessions were supervised by an oncology nurse or exercise physiologist at the exercise clinic of Karolinska University Hospital. All exercise sessions commenced with a warm-up (5 min) on a cycle ergometer or treadmill at a rating of perceived exertion (RPE) of 10–12 on the Borg scale [14] and ended with a cool-down (10 min) consisting of dynamic muscle stretching. The exercise sessions of the AT-HIIT-group started with 20 min of continuous aerobic exercise at a RPE of 13–15 (i.e. moderate intensity) on a cycle ergometer, an elliptical ergometer, or a treadmill. This was followed by 3 × 3 min bouts of intermittent aerobic exercise at a RPE of 16–18 (i.e. high intensity) on a cycle ergometer interspersed with 1 min of low-intensity active recovery. The RT-HIIT-group completed 8 resistance exercises as well as the same high intensity intermittent aerobic exercise as AT-HIIT during each session. The resistance training component consisted of exercises targeting the major muscle groups and included leg press, biceps curls, squat jumps, triceps extensions, lunges, bench press, sit-ups or Russian-weighted abdominal twists, shoulder press, and prone-lying back extensions. Participants completed 2–3 sets of 8–12 repetitions at an initial intensity of 70% of the patients’ 1 repetition maximum (1-RM) strength, increasing to 80% of the estimated 1-RM when more than 12 repetitions could be correctly performed. To ensure overload, new estimated 1-RM tests were performed when participants were able to perform more than 12 repetitions of their 80% 1-RM The UC-group received written information about exercise recommendations for patients with cancer according to the American College of Sports Medicine guidelines [15].

### Data collection

The participant’s SOC was measured at baseline (1 week prior to the second chemotherapy session), whereas the endpoints fatigue, QoL, and symptom burden were measured at baseline and at 16 weeks.

### Sense of coherence

SOC was measured using the Swedish version of Antonovsky’s short 13-item questionnaire [9]. Three SOC dimensions are included: comprehensibility, manageability, and meaningfulness. Participants scored each item on a seven-point Likert scale, where 1 and 7 reflected extreme feelings about statements about how one’s life is experienced. One total SOC score was calculated, ranging from 13 to 91 and a higher score indicated stronger SOC [16]. This score was divided into three categories according to Langius et al. (2007): weak (score ranging between 13 and 45), normal (46–74), and strong SOC score (75–91) [17]. The categories weak and normal SOC were combined due to too few people with weak SOC. The SOC-13 questionnaire is a valid and reliable self-report instrument with high internal consistency (Cronbach’s α ranging from 0.70 to 0.92) [18].

### Outcome measures

Session attendance data were collected from exercise training logs completed by the exercise physiologist or oncology nurse. *Session attendance* was calculated by dividing the number of exercise sessions attended by the number of exercise sessions scheduled. Since *session attendance* was not normally distributed, it was dichotomized based on the median to facilitate meaningful interpretation. *Drop-out from the exercise intervention* was defined as any participant leaving the exercise intervention 1 month prior to post-intervention testing, but showing up at the post-intervention test. *Long-term absence during the intervention period* was defined as any participant missing more than 4 consecutive weeks of the exercise intervention, but coming back to the exercise sessions prior to post-intervention testing. *Drop-out from the study* was defined as any participant leaving the exercise intervention after baseline and not showing up at the post-intervention test.

Cancer-related fatigue was self-assessed using the validated Swedish version of the Piper Fatigue Scale [19, 20]. This 22-item questionnaire was used to subjectively measure four dimensions of fatigue: behaviour/daily life, emotional/affective, sensory/physical, and cognitive. Each item is scored on a scale from 0 to 10, with a low score indicating a low level of fatigue.

Health-related Quality of Life (HRQoL) was assessed using the global quality of life subscale of the validated European Organization for Research and Treatment of Cancer Quality of Life questionnaire (EORTC QLQ-C30) [21].

Distress, severity, and frequency of 32 physical and psychological cancer-related symptoms were assessed using the validated Swedish version of the Memorial Symptom Assessment Scale (MSAS) [22, 23]. The total symptom score was calculated by taking the average of all 32 symptoms, with higher scores indicating greater frequency, more severity, and higher distress.

### Statistical analysis

Statistical analyses were performed using SPSS statistics version 21.0. Sample size calculations were performed for the original OptiTrain study with fatigue as the primary outcome measure. In order to detect a medium effect size of 0.53, and anticipating an attrition rate of ~ 20%, a sample size of 80 participants per group was required (alpha = 0.05, power = 0.80). Due to loss to follow-up during the intervention period and stratification for the SOC level, we were only able to detect large effects.

Characteristics of the whole study population were summarized using descriptive statistics. In addition, these characteristics were stratified for weak-normal and strong SOC. All analyses were performed according to the intention-to-treat principle and missing values were replaced using the expectation-maximization method [24]. The expectation-maximization method was based on group change and the individual baseline score. Since this method requires a baseline score, all participants who were randomized and turned up at baseline testing were included in the analyses. Participants that dropped out after baseline testing and did not complete the baseline questionnaires were excluded from the analysis (*n* = 2). The modified Poisson regression (i.e., Poisson regression with a robust error variance) procedure [25, 26] was applied to determine the relative risk (RR) of low exercise session attendance, study and intervention drop-out, and long-absence rates for patients with weak-normal SOC relative to that of patients with strong SOC.

Analyses of covariance (ANCOVA) analyses were performed to assess the effect of the exercise intervention on fatigue, QoL and symptom burden, controlled for baseline scores of the outcome, chemotherapy treatment (taxanes/no taxanes), and menopausal status. If appropriate, the Bonferroni post hoc test for multiple comparisons was conducted to confirm where the differences occurred between groups. To test whether SOC moderated the exercise intervention effects, an interaction term was added to the model. SOC was considered a potential moderator when the *p* value of the interaction term was ≤ 0.10.

Standardized effect sizes (ES) were calculated by dividing the between-group difference of the post-intervention mean by the pooled baseline standard deviation. According to Cohen, ESs of < 0.2, 0.2–0.5, 0.5–0.8, and > 0.8 indicate no, small, medium, and large effects, respectively [27]. Statistical significance was set at a probability of *p* < 0.05 for all analyses, except for the interaction term.

## Results

### Participants

In total, 240 women signed informed consent and 182 (76%) participants completed both baseline and follow-up testing (Appendix 1) [12]. Two hundred and forty women with breast cancer undergoing chemotherapy were randomly allocated to either AT-HIIT (*n* = 80), RT-HIIT (*n* = 79), or UC (*n* = 81). Participants who did and those who did not complete the study and were comparable with regard to all baseline characteristics (e.g. age, education, menopausal status, and tumour profile) *(p* > 0.05*).*

Baseline demographic, clinical, and psychosocial characteristics, stratified for SOC, are presented in Table 1. Mean total SOC score was 70.4 (standard deviation (SD) = 10.5, range 26–91). Five participants (2.4%) reported low SOC, whereas 120 (58.3%) and 81 (39.3%) participants reported normal and strong SOC, respectively. In this study population, the internal consistency of the SOC-scale was adequate; Cronbach’s α = 0.85.

**Table 1:** Participant characteristics at baseline

	Participants with weak-normal SOC (*n* = 125)	Participants with strong SOC (*n* = 81)	
	Mean ± SD	Mean ± SD	*p* value^a^
Age (years)	52.6 ± 10.6	54.3 ± 9.0	0.23
Body weight (kg)	68.9 ± 11.1	67.8 ± 12.9	0.52
Height (cm)	165.4 ± 7.1	166.4 ± 6.0	0.30
BMI (kg/m^2^)	25.2 ± 4.0	24.5 ± 4.7	0.24
Symptom burden	0.9 ± 0.7	0.6 ± 0.6	< 0.01
Global HRQoL (QLQ-C30)	62.1 ± 22.4	72.4 ± 22.2	< 0.01
Fatigue	3.0 ± 3.0	1.8 ± 2.6	< 0.01
	*n (%)*	*n (%)*	
Married or partnered	65 (52.0)	57 (70.4)	0.09
Education level completed			0.01
Primary school	28 (22.4)	5 (6.2)	
Secondary school	18 (14.4)	16 (19.8)	
Tertiary education	72 (57.6)	59 (72.8)	
Current smoker	7 (5.6)	3 (3.7)	0.58
Menopausal status			0.52
Premenopausal	48 (38.4)	36 (44.4)	
Postmenopausal	76 (60.8)	45 (55.6)	
Tumour profile			0.04
Triple negative	25 (20.0)	4 (4.9)	
HER2+, ER+, PR+	12 (9.6)	13 (16.0)	
HER2+, ER-, PR-	9 (7.2)	5 (6.2)	
HER2-, ER+, PR+	61 (48.8)	45 (55.6)	
HER2-, ER+, PR-	12 (9.6)	8 (9.9)	
HER2+, ER+, PR-	6 (4.8)	4 (4.9)	
HER2-, ER-, PR+	0 (0.0)	2 (2.5)	
Chemotherapy regimen			0.60
Anthracycline	46 (36.8)	35 (43.2)	
Taxane	2 (1.6)	4 (4.9)	
Anthracycline + Taxane	48 (38.4)	25 (30.9)	
Anthracycline + Taxane + Herceptin	28 (22.4)	16 (19.8)	
Anthracycline + Herceptin	1 (0.8)	1 (1.2)	
Group allocation			0.20
RT-HIIT	41 (32.8)	33 (40.7)	
AT-HIIT	42 (33.6)	30 (37.0)	
UC	42 (33.6)	18 (22.2)	

At baseline, characteristics of participants with strong SOC and weak-normal SOC were comparable, except that participants with strong SOC were more often married or partnered (70.4% vs. 52.0%) and had a higher education (72.8% vs. 57.6%). Moreover, on average, they reported significantly less fatigue (− 1.26, 95% confidence interval (CI) − 2.06; − 0.45), lower symptom burden (− 0.39, 95% CI − 0.58; − 0.20), and higher QoL (10.36, 95% CI 4.11; 16.61).

### The risk of low session attendance, dropout, and long-absence

Participants with weak-normal SOC were less likely to have high session attendance (RR = 0.94, 95% CI 0.85; 1.03), albeit not significant (Table 2). Patients with weak-normal SOC had 2.35 times the risk of dropping out from the study (95% CI 1.00; 5.55) compared to patients with strong SOC, whereas the relative risk for intervention drop-out and long-absence was comparable between the two SOC groups (RR = 1.21, 95% CI 0.60;2.56 and RR = 0.87, 95% CI 0.40;1.89, respectively).

**Table 2:** The risk of low session attendance, drop-out, and long absence among women with breast cancer with weak-normal SOC relative to that of women with strong SOC

		Sense of coherence		
		Weak-normal (*n*)	Strong (*n*)	RR (95% CI)
Session attendance^a^	Low (< 15 sessions)	26	38	0.94 (0.85;1.03)
	High (≥ 15 sessions)	18	43	
Study drop-out^b^	Yes	22	6	2.35 (1.00;5.55)
	No	106	76	
Intervention drop-out^a^	Yes	14	11	1.21 (0.60;2.46)
	No	50	50	
Long-absence^a^	Yes	10	11	0.87 (0.40;1.89)
	No	54	50	

*RR* risk ratio, *CI* confidence interval

^a^The analysis included participants who completed the study and were randomized to RT-HIIT or AT-HIIT (*n* = 125)

^b^Participants who did not drop out before baseline (*n* = 210)

### SOC as moderator of exercise effects on fatigue, QoL, and symptom burden

In this paper, both the overall intervention effects and the effects stratified by the potential moderator SOC are presented in Table 3.

**Table 3:** Exercise intervention effects on fatigue, total symptoms, symptom burden and QoL, stratified by sense of coherence

	Adjusted mean between-group difference (95% CI)		*p*_interaction (intervention × SOC)_	Effect size	
	RT-HIIT vs UC^a^	AT-HIIT vs UC^a^		RT-HIIT vs UC	AT-HIIT vs UC
Cancer-related fatigue (PFS)					
Total CRF^b^	− 1.17 (− 2.18; − 20.16)*	− 0.72 (− 1.73; 0.30)	0.88	0.39	0.25
Weak-normal SOC	− 1.09 (− 2.30; 0.13)	− 0.87 (− 2.10; 0.36)		0.36	0.30
Strong SOC	− 1.01 (− 2.90; 0.88)	− 0.44 (− 2.32; 1.45)		0.39	0.21
Sensory/physical CRF^b^	− 1.22 (− 2.33; − 20.11)*	− 0.64 (− 1.76; 0.49)	0.68	0.38	0.21
Weak-normal SOC	− 1.16 (− 2.52; 0.20)	− 0.95 (− 2.32; 0.42)		0.35	0.29
Strong SOC	− 0.97 (− 2.98; 1.04)	− 0.12 (− 2.14; 1.89)		0.35	0.05
Behaviour/daily life CRF^b^	− 1.46 (− 2.55; − 20.37)*	− 0.96 (− 2.05; 0.14)	0.98	0.47	0.36
Weak-normal SOC	− 1.46 (− 2.78; − 20.14)*	− 1.07 (− 2.38; 0.25)		0.45	0.37
Strong SOC	− 1.24 (− 3.27; 0.79)	− 0.80 (− 2.82; 1.23)		0.49	0.40
Emotional/affective CRF^b^	− 1.10 (− 2.26; 0.06)	− 0.49 (− 1.66; 0.68)	0.69	0.34	0.16
Weak-normal SOC	− 0.78 (− 2.14; 0.58)	− 0.60 (− 1.97; 0.77)		0.24	0.19
Strong SOC	− 1.37 (− 3.60; 0.86)	− 0.36 (− 2.60; 1.87)		0.45	0.14
Cognitive CRF^b^	− 0.89 (− 1.81; 0.03)	− 0.70 (− 1.63; 0.23)	0.91	0.31	0.27
Weak-normal SOC	− 0.91 (− 2.04; 0.22)	− 0.78 (− 1.92; 0.36)		0.31	0.28
Strong SOC	− 0.46 (−2.14; 1.22)	− 0.39 (− 2.06; 1.28)		0.19	0.21
Symptoms (MSAS)					
Total symptoms^b^	− 0.20 (− 0.37; − 20.04)*	− 0.13 (− 0.30; 0.03)	0.10	0.39	0.29
Weak-normal SOC	− 0.24 (− 0.46; − 20.02)*	− 0.05 (− 0.27; 0.17)		0.44	0.11
Strong SOC	− 0.07 (− 0.32; 0.18)	− 0.15 (− 0.40; 0.10)		0.19	0.40
Symptom burden^b^	− 0.22 (− 0.41; − 20.02)*	− 0.24 (− 0.43; − 0.04)*	0.27	0.30	0.37
Weak-normal SOC	− 0.24 (− 0.48; 0.01)	− 0.13 (− 0.38; 0.11)		0.31	0.21
Strong SOC	− 0.10 (− 0.43; 0.22)	− 0.26 (− 0.49; 0.07)		0.21	0.47
Health-related quality of life (EORTC-QLQ-C30)					
Global health status/QoL^b^	5.47 (−1.74; 12.67)	4.67 (− 2.60; 11.94)	0.19	0.23	0.22
Weak-normal SOC	5.71 (−3.80; 15.21)	1.00 (− 8.62; 10.62)		0.25	0.05
Strong SOC	4.33 (−7.29; 15.95)	9.41 (− 2.28; 21.10)		0.19	0.51

*CI* confidence interval, *PFS* Piper Fatigue Scale, *CRF* cancer-related fatigue, *MSAS* Memorial Symptom Assessment Scale, *QoL* quality of life

^a^RT-HIIT: overall *n* = 74, weak-normal SOC *n* = 41, strong SOC *n* = 33. AT-HIIT: overall *n* = 70, weak-normal SOC *n* = 40, strong SOC *n* = 30

*UC* overall *n* = 60, weak-normal SOC *n* = 42, strong SOC *n* = 18. ^b^Total effects of the exercise interventions on fatigue, symptoms, and health-related quality of life have been previously reported [12]

At 16 weeks, participants randomized to the RT-HIIT-group reported significantly lower total fatigue levels compared to participants randomized to the UC-group (− 1.17, 95% CI − 2.18; − 0.16, ES = 0.39), whereas there was no significant difference between AT-HIIT and UC. Similar results were found for the fatigue dimensions: behaviour/daily life and sensory/physical. No statistically significant moderating effects were found for SOC (*p*_interaction_ = 0.88), indicating that the exercise intervention effects on fatigue were comparable for participants with weak-normal SOC and strong SOC.

A non-significant greater magnitude of change in QoL during the intervention was found for participants in the RT-HIIT-group (5.47, 95% CI − 1.47; 12.67, ES = 0.23) and AT-HIIT-group (4.67, 95% CI − 2.60; 11.94, ES = 0.19) compared to the UC-group. No statistically significant moderating effect was found (*p*_interaction_ = 0.19).

Participants who participated in RT-HIIT reported significantly less symptoms (− 0.20, 95% CI − 0.37; − 0.04, ES = 0.39), and lower symptom burden over the 16-week intervention (− 0.22, 95% CI − 0.41; − 0.02, ES = 0.30) compared to participants in the UC group, whereas there was no significant difference between AT-HIIT and UC. An interaction effect of SOC was found (*p*_interaction_ = 0.10), indicating that the effect of RT-HIIT on total symptoms was larger for participants with weak-normal SOC (− 0.24, 95% CI − 0.46; − 0.02, ES = 0.44) than for those with strong SOC (− 0.07, 95% CI − 0.32;0.18, ES = 0.19), whereas the effect of AT-HIIT on total symptoms was larger for participants with strong SOC (− 0.15, 95% CI − 0.40; 0.10, ES = 0.40) than for those with weak-normal SOC (− 0.05, 95% CI − 0.27;0.17, ES = 0.11).

## Discussion

In the current study, the majority of women participating in the exercise trial had weak-normal SOC (61%). Women with strong SOC were more often married and highly educated. In addition, they experienced significantly less fatigue, reported a lower symptom burden and higher QoL. These results support Antonovsky’s salutogenic theory [9] that those with stronger SOC are able to successfully manage stressors to maintain health. This study shows that women with weak-normal SOC were more likely to drop out from the study and tended to have slightly poorer exercise session attendance. Furthermore, our results indicated that women with breast cancer and weaker SOC benefitted as much from the exercise intervention as those with stronger SOC in terms of fatigue and QoL. Surprisingly, SOC moderated the effect of RT-HIIT on total symptoms in favour of participants with weak-normal SOC. This moderating effect of SOC can be partially explained by the higher score on total symptoms in participants with weak-normal SOC allowing for larger changes over time.

In the current study, the mean total SOC score was 70.4 (SD = 10.5), which is in accordance with one other study in women with breast cancer [28], whereas another study in patients with mainly breast cancer demonstrated slightly lower scores, approximately 7 points less [29]. When comparing the distribution of SOC in the Swedish population to this study population, the distribution includes a larger proportion of people with strong SOC (33% vs. 39% individuals with strong SOC, respectively) [30].

It has been shown that SOC is positively associated with perceived health [11]. The results from the current study indicate that SOC plays a significant role in how women with breast cancer experience cancer-related fatigue, symptom burden, and QoL, which is in line with previous studies including other populations. Several studies reported that perceived health, distress, symptom burden, and QoL can be predicted by SOC and that people with stronger SOC are more likely to develop a positive subjective state of health [11, 28, 31–34].

Congruent with the hypothesis that those with stronger SOC may more actively pursue health-promoting activities [11] and thus, may more actively engage in exercise interventions, our results indicated that weaker SOC was associated with a slightly poorer session attendance and a higher study drop-out rate. Similar associations were found in the elderly with a hip fracture history [35]. French et al. (2006) proposed that individuals who view their condition as uncontrollable may lack an understanding of the purpose of an exercise intervention [36]. It is therefore of importance that in clinical practice, healthcare professionals are able to individualize physical activity recommendations based on the patients’ preferences, barriers, and abilities in order to support women with weak SOC to engage in physical activity [37, 38].

Unfortunately, no data on SOC was available for eligible patients who were invited but declined to participate in the OptiTrain study. Previous studies found that these non-participants had lower education, a less positive attitude towards exercise, lower perception of self-efficacy, reported more fatigue, lower QoL, a lower pre-diagnosis physical activity level, and perceived more barriers to exercising [39–41]. Hence, non-participants may be the individuals that are in the greatest need of an exercise intervention. For future studies, it might be interesting to get insight into the SOC of those (non)-participants since it allows for an identification of patients who are at risk of drop-out and poor adherence. It will also allow for a better understanding of the bias that might be introduced by selective drop-out. In addition, it provides information about how we might adapt future exercise interventions to specifically target patients who are less inclined to participate.

Limitations of the study include the small sample size, which did not allow for valid comparisons between the two exercise intervention groups, stratified for the SOC level. Second, due to the characteristics of this study population, generalization to other populations should be done with caution. Finally, despite the longitudinal design, conclusions about causality should be drawn with caution and need to be confirmed in future studies.

## Conclusion

A sense of coherence might have a positive effect on perceived health in terms of cancer-related fatigue, symptom burden, and QoL. We found that strong SOC was related to lower levels of cancer-related fatigue, enhanced QoL, and lower symptom burden in women with breast cancer receiving chemotherapy. Furthermore, we can conclude that participants with weak-normal and strong SOC benefit equally from the exercise intervention. While women with weak-normal SOC may not need additional support in physical exercise programs to optimize training response, they may need support to take the step to participate and adhere to exercise trials. The SOC scale may operate as a tool to identify participants with weak SOC. This will aid in developing strategies to reach out to those who are unwilling to participate and to incorporate more motivational efforts for those who are likely to drop out from an exercise study in order to get a more representative sample. Finally, assessing SOC may assist health care professionals in providing more effective individualized exercise interventions.

## Electronic supplementary material

ESM 1 CONSORT diagram. Participant flow through the study. *RT–HIIT* resistance and high-intensity interval training, *AT–HIIT* moderate-intensity aerobic and high-intensity interval training, *UC* usual care (PDF 140 kb)
