# Supplementary material for: Sense of coherence and its relationship to participation, cancer-related fatigue, symptom burden, and quality of life in women with breast cancer participating in the OptiTrain exercise trial
Source: Support Care Cancer. 2020 Mar 5;28(11):5371–9. doi: 10.1007/s00520-020-05378-0 (PMC7546973; doi:10.1007/s00520-020-05378-0)
Supplement: Supplementary file 1 — CONSORT diagram. Participant flow through the study. RT–HIIT resistance and high-intensity interval training, AT–HIIT moderate-intensity aerobic and high-intensity interval training, UC usual care (PDF 140 kb) [file 520_2020_5378_MOESM1_ESM.pdf]

Title Manuscript: Sense of coherence and its relationship to participation, cancer-related fatigue, symptom burden and quality of life in women with breast cancer participating in the OptiTrain exercise trial

Anouk E Hiensch[*,1], Kate A Bolam[2,3], Sara Mijwel[2,4], Anne M May[1], Yvonne Wengström[2,5]

[1]Julius Center for Health Sciences and Primary Care, University Medical Center Utrecht, PO Box 85500, STR 6.131, 3508 GA Utrecht, The Netherlands.
[2]Department of Neurobiology, Care Sciences and Society, Division of Nursing, Karolinska Institutet, Karolinska University Hospital, 141 83 Huddinge, Stockholm, Sweden.
[3]School of Human Movement and Nutrition Sciences, The University of Queensland, Brisbane, Australia.
[4]Department of Physiology and Pharmacology, Karolinska Institutet, Stockholm, Sweden.
[5]Theme Cancer, Karolinska University Hospital, Stockholm, Sweden.

* Corresponding author: A.E. Hiensch, Julius Center for Health Sciences and Primary Care, University Medical Center Utrecht, Julius Centrum, PO Box 85500, STR 6.131, 3508 GA Utrecht, The Netherlands. Tel: +31(0)88 75 677 08, Email: a.e.hiensch-2@umcutrecht.nl, (ORCID: 0000-0002-6216-2609)

Journal of Supportive Care in Cancer

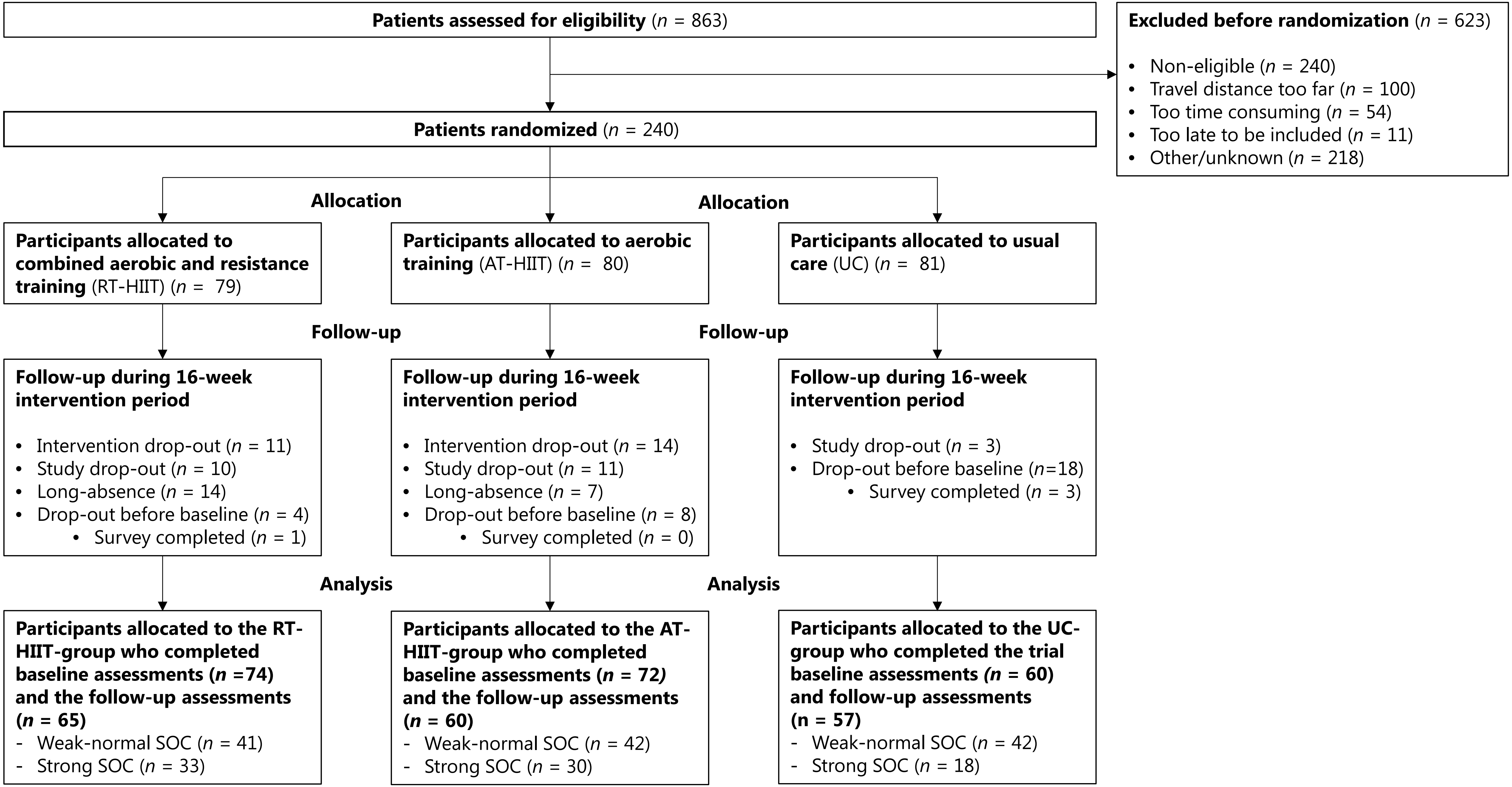
Patients assessed for eligibility (n = 863)
Excluded before randomization (n = 623)
• Non-eligible (n = 240)
• Travel distance too far (n = 100)
• Too time consuming (n = 54)
• Too late to be included (n = 11)
• Other/unknown (n = 218)
Patients randomized (n = 240)
Allocation
Allocation
Participants allocated to combined aerobic and resistance training (RT-HIIT) (n = 79)
Participants allocated to aerobic training (AT-HIIT) (n = 80)
Participants allocated to usual care (UC) (n = 81)
Follow-up
Follow-up
Follow-up during 16-week intervention period
• Intervention drop-out (n = 11)
• Study drop-out (n = 10)
• Long-absence (n = 14)
• Drop-out before baseline (n = 4)
• Survey completed (n = 1)
Follow-up during 16-week intervention period
• Intervention drop-out (n = 14)
• Study drop-out (n = 11)
• Long-absence (n = 7)
• Drop-out before baseline (n = 8)
• Survey completed (n = 0)
Follow-up during 16-week intervention period
• Study drop-out (n = 3)
• Drop-out before baseline (n=18)
• Survey completed (n = 3)
Analysis
Analysis
Participants allocated to the RT-HIIT-group who completed baseline assessments (n =74) and the follow-up assessments (n = 65)
- Weak-normal SOC (n = 41)
- Strong SOC (n = 33)
Participants allocated to the AT-HIIT-group who completed baseline assessments (n = 72) and the follow-up assessments (n = 60)
- Weak-normal SOC (n = 42)
- Strong SOC (n = 30)
Participants allocated to the UC-group who completed the trial baseline assessments (n = 60) and follow-up assessments (n = 57)
- Weak-normal SOC (n = 42)
- Strong SOC (n = 18)
